# 4-(4-Chloro-5-methyl-3-trifluoro­meth­yl-1*H*-pyrazol-1-yl)-6-(prop-2-ynyl­oxy)pyrimidine

**DOI:** 10.1107/S1600536810031740

**Published:** 2010-08-18

**Authors:** Ru-Liang Xie, Tao Zhang, Ao-Cheng Cao, Xiang-Dong Mei

**Affiliations:** a State Key Laboratory for the Biology of Plant Diseases and Insect Pests, Institute of Plant Protection, Chinese Academy of Agricultural Sciences, Beijing 100193, People’s Republic of China

## Abstract

The molecule of the title compound, C_12_H_8_ClF_3_N_4_O, is twisted as indicated by the C—O—C—C torsion angle of 76.9 (3)°. Moreover, the trifluoro­methyl group shows rotational disorder of the F atoms, with site-occupancy factors of 0.653 (6) and 0.347 (6). The dihedral angle between the rings is 1.88 (12) Å.

## Related literature

For the applications of pyrazole derivatives, see: Hirai *et al.* (2002 ▶); Krishnaiah *et al.* (2002 ▶); Ohno *et al.* (2004 ▶); Li *et al.* (2008 ▶); Shiga *et al.* (2003 ▶); Vicentini *et al.* (2007 ▶).
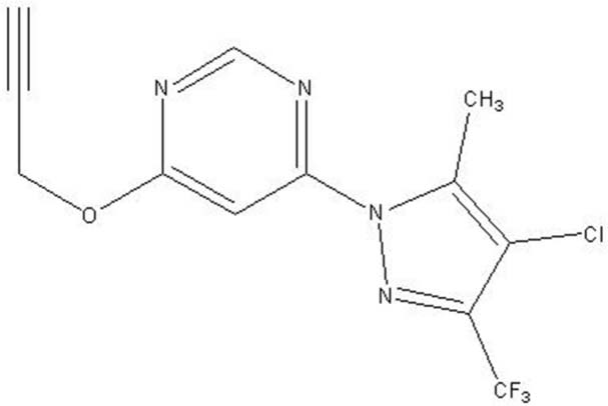


## Experimental

### 

#### Crystal data

C_12_H_8_ClF_3_N_4_O
*M* *_r_* = 316.67
Monoclinic, 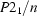

*a* = 7.8331 (13) Å
*b* = 7.7258 (12) Å
*c* = 21.757 (4) Å
β = 99.270 (11)°
*V* = 1299.5 (4) Å^3^
*Z* = 4
Cu *K*α radiation
μ = 3.02 mm^−1^
*T* = 173 K
0.20 × 0.20 × 0.10 mm

#### Data collection

Rigaku R-AXIS RAPID IP area-detector diffractometer
Absorption correction: multi-scan (*ABSCOR*; Higashi, 1995 ▶) *T*_min_ = 0.583, *T*_max_ = 0.752
8543 measured reflections
2361 independent reflections
2009 reflections with *I* > 2σ(*I*)
*R*_int_ = 0.043

#### Refinement

*R*[*F* ^2^ > 2σ(*F* ^2^)] = 0.044
*wR*(*F* ^2^) = 0.106
*S* = 1.07
2361 reflections
220 parameters
69 restraints
H-atom parameters constrained
Δρ_max_ = 0.31 e Å^−3^
Δρ_min_ = −0.22 e Å^−3^

### 

Data collection: *RAPID-AUTO* (Rigaku, 2001 ▶); cell refinement: *RAPID-AUTO*; data reduction: *RAPID-AUTO*; program(s) used to solve structure: *SHELXS97* (Sheldrick, 2008 ▶); program(s) used to refine structure: *SHELXL97* (Sheldrick, 2008 ▶); molecular graphics: *XP* (Siemens, 1998 ▶); software used to prepare material for publication: *SHELXL97*.

## Supplementary Material

Crystal structure: contains datablocks I, global. DOI: 10.1107/S1600536810031740/rn2068sup1.cif

Structure factors: contains datablocks I. DOI: 10.1107/S1600536810031740/rn2068Isup2.hkl

Additional supplementary materials:  crystallographic information; 3D view; checkCIF report

## Figures and Tables

**Fig. 1.**
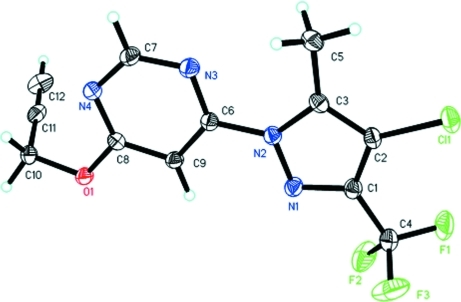
The structure of the title compound with labeling scheme; displacement ellipsoids are shown at the 30% probability level and atoms F1', F2' and F3' representing the smaller fraction of the disordered trifluoromethyl group have been excluded.

## supplementary crystallographic information

### Comment

There is much agrochemical interest in pyrazole derivatives because of their excellent bioactivity (Krishnaiah *et al.*, 2002; Ohno *et al.*, 2004; Li *et al.*, 2008 Shiga *et al.*, 2003; Vicentini *et al*., 2007). Numerous herbicides such as pyrazolate, pyrazoxyfen, benzofenap, pyraflufen-ethyl, fluazolate and pyrazosulfuron-ethyl with pyrazole moieties were commercialized (Hirai *et al.*, 2002). Recently, a novel pyrazole derivative (I) with a trifluoromethyl group was synthesized. The trifluoromethyl group shows rotational disorder of the F atoms, with site occupancy factors of 0.653 (6) and 0.347 (6). This molecule is twisted, prop-2-ynyloxy is out of the pyrimidine ring plane, as indicated by the C(8)—O(1)—C(10)—C(11) torsion angle of 76.9 (3)°. The crystal structure of the title compound is shown in Fig. 1.

### Experimental

The title compound (0.15 g) was dissolved in the mixed solvent of ethanol and acetone (25 mL) at room temperature. Colorless single crystals of compound (I) were obtained through slow evaporation after two weeks.

### Refinement

The trifluoromethyl group shows rotational disorder of the F atoms, with site occupancy factors of 0.653 (6) and 0.347 (6).All the hydrogen atoms were placed at their geometrical position with C—H = 0.93-0.98Å and *U*_iso_(H) = 1.2-1.5*U*_eq_(C).

### Crystal data

**Table:**

C_12_H_8_ClF_3_N_4_O	*F*(000) = 640
*M**_r_* = 316.67	*D*_x_ = 1.619 Mg m^−^^3^
Monoclinic, *P*2_1_/*n*	Cu *K*α radiation, λ = 1.54186 Å
*a* = 7.8331 (13) Å	Cell parameters from 564 reflections
*b* = 7.7258 (12) Å	θ = 2.2–68.3°
*c* = 21.757 (4) Å	µ = 3.02 mm^−^^1^
β = 99.270 (11)°	*T* = 173 K
*V* = 1299.5 (4) Å^3^	Platelet, colorless
*Z* = 4	0.20 × 0.20 × 0.10 mm

### Data collection

**Table:**

Rigaku R-AXIS RAPID IP area-detector diffractometer	2361 independent reflections
Radiation source: rotating anode	2009 reflections with *I* > 2σ(*I*)
graphite	*R*_int_ = 0.043
ω scans at fixed χ = 45°	θ_max_ = 68.3°, θ_min_ = 4.1°
Absorption correction: multi-scan (*ABSCOR*; Higashi, 1995)	*h* = −9→9
*T*_min_ = 0.583, *T*_max_ = 0.752	*k* = −9→6
8543 measured reflections	*l* = −26→22

### Refinement

**Table:**

Refinement on *F*^2^	Secondary atom site location: difference Fourier map
Least-squares matrix: full	Hydrogen site location: inferred from neighbouring sites
*R*[*F*^2^ > 2σ(*F*^2^)] = 0.044	H-atom parameters constrained
*wR*(*F*^2^) = 0.106	*w* = 1/[σ^2^(*F*_o_^2^) + (0.0399*P*)^2^ + 0.8523*P*] where *P* = (*F*_o_^2^ + 2*F*_c_^2^)/3
*S* = 1.07	(Δ/σ)_max_ = 0.016
2361 reflections	Δρ_max_ = 0.31 e Å^−^^3^
220 parameters	Δρ_min_ = −0.22 e Å^−^^3^
69 restraints	Extinction correction: *SHELXL97* (Sheldrick, 2008), Fc^*^=kFc[1+0.001xFc^2^λ^3^/sin(2θ)]^-1/4^
Primary atom site location: structure-invariant direct methods	Extinction coefficient: 0.0054 (5)

### Special details

**Table:**

Geometry. All esds (except the esd in the dihedral angle between two l.s. planes) are estimated using the full covariance matrix. The cell esds are taken into account individually in the estimation of esds in distances, angles and torsion angles; correlations between esds in cell parameters are only used when they are defined by crystal symmetry. An approximate (isotropic) treatment of cell esds is used for estimating esds involving l.s. planes.
Refinement. Refinement of *F*^2^ against ALL reflections. The weighted *R*-factor wR and goodness of fit *S* are based on *F*^2^, conventional *R*-factors *R* are based on *F*, with *F* set to zero for negative *F*^2^. The threshold expression of *F*^2^ > σ(*F*^2^) is used only for calculating *R*-factors(gt) etc. and is not relevant to the choice of reflections for refinement. *R*-factors based on *F*^2^ are statistically about twice as large as those based on *F*, and *R*- factors based on ALL data will be even larger.

### Fractional atomic coordinates and isotropic or equivalent isotropic displacement parameters (Å^2^)

**Table:**

	*x*	*y*	*z*	*U*_iso_*/*U*_eq_	Occ. (<1)
Cl1	0.05485 (8)	0.66461 (9)	0.12912 (3)	0.0419 (2)	
F1	0.0581 (14)	1.0026 (19)	0.2067 (6)	0.068 (3)	0.65 (3)
F2	0.1586 (17)	1.2382 (8)	0.1787 (5)	0.066 (2)	0.65 (3)
F3	0.3301 (10)	1.040 (2)	0.2184 (4)	0.080 (3)	0.65 (3)
F1'	0.038 (2)	1.038 (4)	0.2018 (12)	0.062 (4)	0.35 (3)
F2'	0.229 (4)	1.227 (2)	0.1908 (8)	0.074 (4)	0.35 (3)
F3'	0.298 (3)	0.977 (3)	0.2215 (7)	0.076 (4)	0.35 (3)
O1	0.4744 (2)	1.4528 (2)	−0.08155 (7)	0.0328 (4)	
N1	0.2473 (2)	1.0968 (3)	0.07612 (9)	0.0307 (5)	
N2	0.2397 (2)	0.9953 (2)	0.02454 (9)	0.0273 (4)	
N3	0.2814 (3)	0.9661 (3)	−0.07855 (9)	0.0339 (5)	
N4	0.4008 (2)	1.1943 (3)	−0.13275 (9)	0.0300 (5)	
C1	0.1864 (3)	0.9977 (3)	0.11729 (11)	0.0298 (5)	
C2	0.1401 (3)	0.8333 (3)	0.09272 (11)	0.0292 (5)	
C3	0.1757 (3)	0.8320 (3)	0.03301 (11)	0.0280 (5)	
C4	0.1845 (3)	1.0661 (4)	0.18093 (13)	0.0407 (6)	
C5	0.1542 (3)	0.6869 (3)	−0.01239 (12)	0.0369 (6)	
H5A	0.2630	0.6681	−0.0282	0.055*	
H5B	0.0626	0.7155	−0.0471	0.055*	
H5C	0.1228	0.5814	0.0082	0.055*	
C6	0.2966 (3)	1.0682 (3)	−0.02856 (10)	0.0261 (5)	
C7	0.3362 (3)	1.0361 (3)	−0.12775 (12)	0.0349 (6)	
H7A	0.3284	0.9648	−0.1637	0.042*	
C8	0.4111 (3)	1.2901 (3)	−0.08172 (11)	0.0271 (5)	
C9	0.3614 (3)	1.2338 (3)	−0.02663 (10)	0.0275 (5)	
H9A	0.3714	1.3042	0.0096	0.033*	
C10	0.5077 (3)	1.5223 (3)	−0.14053 (11)	0.0350 (6)	
H10A	0.5662	1.4332	−0.1624	0.042*	
H10B	0.5861	1.6231	−0.1325	0.042*	
C11	0.3481 (3)	1.5756 (3)	−0.18034 (11)	0.0355 (6)	
C12	0.2244 (4)	1.6239 (4)	−0.21331 (13)	0.0516 (8)	
H12	0.1240	1.6632	−0.2401	0.062*	

### Atomic displacement parameters (Å^2^)

**Table:**

	*U*^11^	*U*^22^	*U*^33^	*U*^12^	*U*^13^	*U*^23^
Cl1	0.0394 (4)	0.0400 (4)	0.0474 (4)	−0.0066 (3)	0.0100 (3)	0.0144 (3)
F1	0.087 (5)	0.079 (5)	0.049 (3)	−0.028 (4)	0.042 (4)	−0.006 (3)
F2	0.111 (5)	0.046 (2)	0.048 (3)	0.007 (3)	0.035 (3)	−0.0082 (17)
F3	0.055 (2)	0.137 (6)	0.046 (3)	0.014 (3)	−0.0046 (18)	−0.036 (3)
F1'	0.040 (5)	0.092 (9)	0.058 (6)	0.002 (5)	0.021 (4)	−0.015 (6)
F2'	0.128 (9)	0.058 (5)	0.044 (5)	−0.048 (6)	0.034 (6)	−0.014 (4)
F3'	0.089 (7)	0.092 (8)	0.038 (4)	0.028 (5)	−0.015 (4)	0.006 (5)
O1	0.0436 (10)	0.0302 (9)	0.0243 (9)	−0.0109 (8)	0.0043 (7)	0.0016 (7)
N1	0.0350 (11)	0.0304 (11)	0.0280 (11)	−0.0009 (9)	0.0088 (8)	−0.0017 (8)
N2	0.0286 (10)	0.0263 (10)	0.0275 (10)	−0.0011 (8)	0.0057 (8)	0.0002 (8)
N3	0.0423 (12)	0.0294 (11)	0.0307 (11)	−0.0047 (9)	0.0079 (9)	−0.0037 (9)
N4	0.0310 (10)	0.0307 (11)	0.0288 (11)	−0.0015 (9)	0.0064 (8)	−0.0027 (8)
C1	0.0274 (12)	0.0326 (13)	0.0302 (13)	0.0023 (10)	0.0070 (9)	0.0035 (10)
C2	0.0239 (11)	0.0312 (13)	0.0329 (13)	−0.0002 (10)	0.0058 (9)	0.0075 (10)
C3	0.0220 (11)	0.0263 (12)	0.0350 (13)	0.0006 (9)	0.0023 (9)	0.0026 (10)
C4	0.0404 (14)	0.0476 (17)	0.0370 (15)	−0.0060 (13)	0.0149 (12)	0.0012 (12)
C5	0.0393 (13)	0.0305 (13)	0.0405 (15)	−0.0069 (11)	0.0053 (11)	−0.0012 (11)
C6	0.0237 (11)	0.0276 (12)	0.0269 (12)	0.0016 (9)	0.0040 (9)	0.0013 (9)
C7	0.0447 (14)	0.0318 (14)	0.0292 (13)	−0.0044 (11)	0.0095 (11)	−0.0075 (10)
C8	0.0243 (11)	0.0260 (12)	0.0305 (13)	−0.0012 (9)	0.0032 (9)	0.0005 (10)
C9	0.0320 (12)	0.0254 (12)	0.0248 (12)	−0.0024 (10)	0.0035 (9)	−0.0017 (9)
C10	0.0406 (14)	0.0362 (14)	0.0290 (13)	−0.0099 (11)	0.0078 (10)	0.0053 (11)
C11	0.0497 (15)	0.0310 (14)	0.0269 (13)	0.0004 (12)	0.0093 (11)	−0.0015 (10)
C12	0.0613 (19)	0.0503 (18)	0.0402 (16)	0.0150 (15)	−0.0008 (14)	−0.0051 (14)

### Geometric parameters (Å, °)

**Table:**

Cl1—C2	1.715 (2)	C1—C2	1.403 (3)
F1—C4	1.309 (7)	C1—C4	1.484 (4)
F2—C4	1.344 (6)	C2—C3	1.372 (3)
F3—C4	1.306 (7)	C3—C5	1.486 (3)
F1'—C4	1.316 (12)	C5—H5A	0.9800
F2'—C4	1.300 (11)	C5—H5B	0.9800
F3'—C4	1.339 (10)	C5—H5C	0.9800
O1—C8	1.352 (3)	C6—C9	1.374 (3)
O1—C10	1.452 (3)	C7—H7A	0.9500
N1—C1	1.324 (3)	C8—C9	1.389 (3)
N1—N2	1.362 (3)	C9—H9A	0.9500
N2—C3	1.380 (3)	C10—C11	1.461 (3)
N2—C6	1.420 (3)	C10—H10A	0.9900
N3—C7	1.330 (3)	C10—H10B	0.9900
N3—C6	1.333 (3)	C11—C12	1.170 (4)
N4—C8	1.326 (3)	C12—H12	0.9500
N4—C7	1.334 (3)		
			
C8—O1—C10	117.33 (18)	F3—C4—C1	112.9 (4)
C1—N1—N2	104.57 (19)	F1—C4—C1	112.9 (7)
N1—N2—C3	112.62 (19)	F1'—C4—C1	113.8 (12)
N1—N2—C6	117.62 (18)	F3'—C4—C1	109.1 (8)
C3—N2—C6	129.8 (2)	F2—C4—C1	110.0 (4)
C7—N3—C6	114.7 (2)	C3—C5—H5A	109.5
C8—N4—C7	114.6 (2)	C3—C5—H5B	109.5
N1—C1—C2	111.4 (2)	H5A—C5—H5B	109.5
N1—C1—C4	119.0 (2)	C3—C5—H5C	109.5
C2—C1—C4	129.5 (2)	H5A—C5—H5C	109.5
C3—C2—C1	106.7 (2)	H5B—C5—H5C	109.5
C3—C2—Cl1	125.8 (2)	N3—C6—C9	124.0 (2)
C1—C2—Cl1	127.46 (19)	N3—C6—N2	115.5 (2)
C2—C3—N2	104.7 (2)	C9—C6—N2	120.5 (2)
C2—C3—C5	128.1 (2)	N3—C7—N4	127.9 (2)
N2—C3—C5	127.3 (2)	N3—C7—H7A	116.1
F2'—C4—F3	81.9 (8)	N4—C7—H7A	116.1
F2'—C4—F1	119.3 (10)	N4—C8—O1	119.6 (2)
F3—C4—F1	108.6 (7)	N4—C8—C9	124.1 (2)
F2'—C4—F1'	109.0 (14)	O1—C8—C9	116.3 (2)
F3—C4—F1'	118.7 (11)	C6—C9—C8	114.7 (2)
F1—C4—F1'	14.2 (15)	C6—C9—H9A	122.6
F2'—C4—F3'	104.4 (9)	C8—C9—H9A	122.6
F3—C4—F3'	24.2 (8)	O1—C10—C11	111.7 (2)
F1—C4—F3'	89.7 (10)	O1—C10—H10A	109.3
F1'—C4—F3'	102.3 (13)	C11—C10—H10A	109.3
F2'—C4—F2	25.2 (10)	O1—C10—H10B	109.3
F3—C4—F2	106.6 (5)	C11—C10—H10B	109.3
F1—C4—F2	105.4 (7)	H10A—C10—H10B	107.9
F1'—C4—F2	92.4 (11)	C12—C11—C10	177.0 (3)
F3'—C4—F2	127.7 (7)	C11—C12—H12	180.0
F2'—C4—C1	116.8 (7)		
			
C1—N1—N2—C3	−0.5 (2)	C2—C1—C4—F1'	−49.2 (13)
C1—N1—N2—C6	179.62 (18)	N1—C1—C4—F3'	−112.0 (12)
N2—N1—C1—C2	0.1 (2)	C2—C1—C4—F3'	64.2 (13)
N2—N1—C1—C4	177.0 (2)	N1—C1—C4—F2	32.5 (7)
N1—C1—C2—C3	0.3 (3)	C2—C1—C4—F2	−151.3 (7)
C4—C1—C2—C3	−176.2 (2)	C7—N3—C6—C9	0.6 (3)
N1—C1—C2—Cl1	−179.60 (17)	C7—N3—C6—N2	−179.65 (19)
C4—C1—C2—Cl1	3.9 (4)	N1—N2—C6—N3	−177.90 (19)
C1—C2—C3—N2	−0.6 (2)	C3—N2—C6—N3	2.3 (3)
Cl1—C2—C3—N2	179.31 (16)	N1—N2—C6—C9	1.8 (3)
C1—C2—C3—C5	178.3 (2)	C3—N2—C6—C9	−178.0 (2)
Cl1—C2—C3—C5	−1.8 (4)	C6—N3—C7—N4	−1.0 (4)
N1—N2—C3—C2	0.7 (2)	C8—N4—C7—N3	0.4 (4)
C6—N2—C3—C2	−179.5 (2)	C7—N4—C8—O1	−179.9 (2)
N1—N2—C3—C5	−178.1 (2)	C7—N4—C8—C9	0.6 (3)
C6—N2—C3—C5	1.7 (4)	C10—O1—C8—N4	7.7 (3)
N1—C1—C4—F2'	6.0 (16)	C10—O1—C8—C9	−172.72 (19)
C2—C1—C4—F2'	−177.7 (16)	N3—C6—C9—C8	0.2 (3)
N1—C1—C4—F3	−86.4 (9)	N2—C6—C9—C8	−179.49 (19)
C2—C1—C4—F3	89.9 (9)	N4—C8—C9—C6	−0.9 (3)
N1—C1—C4—F1	150.0 (8)	O1—C8—C9—C6	179.58 (19)
C2—C1—C4—F1	−33.8 (8)	C8—O1—C10—C11	76.9 (3)
N1—C1—C4—F1'	134.5 (13)	O1—C10—C11—C12	149 (6)
